# The evolution of mean arterial pressure in critically ill patients on vasopressors before and during a trial comparing a specific mean arterial pressure target to usual care

**DOI:** 10.1186/s12871-021-01529-w

**Published:** 2022-01-03

**Authors:** Marie-Hélène Masse, Neill K. J. Adhikari, Xavier Théroux, Marie-Claude Battista, Frédérick D’Aragon, Ruxandra Pinto, Alan Cohen, Michaël Mayette, Charles St-Arnaud, Michelle Kho, Michaël Chassé, Martine Lebrasseur, Irene Watpool, Rebecca Porteous, M. Elizabeth Wilcox, François Lamontagne

**Affiliations:** 1 Faculty of Medicine and Health Sciences, Université de Sherbrooke, 12e Avenue Nord, Sherbrooke, Québec J1H 5N4 Canada; 2 Interdepartmental Division of Critical Care Medicine, University of Toronto, 209 Victoria Street, Toronto, Ontario M5B 1T8 Canada; 3 Department of Critical Care Medicine, Sunnybrook Health Sciences Centre, 2075 Bayview Avenue, Toronto, Ontario M4N 3M5 Canada; 4 Faculty of Health Sciences, School of Rehabilitation Science, Institute of Applied Health Sciences, McMaster University, 1280 Main Street West, Hamilton, Ontario L8S 4L8 Canada; 5 Department of Medicine, Université de Montréal, 2900 Boulevard Édouard-Montpetit, Montréal, Québec H3T 1J4 Canada; 6 Centre de recherche, Centre hospitalier de l’Université de Montréal, 900 rue Saint-Denis, Montréal, Québec H2X 0A9 Canada; 7 Ottawa Hospital Research Institute, 1053 Carling Ave, Ottawa, Ontario K1Y 4E9 Canada

**Keywords:** Arterial pressure, Control groups, Critical care, Research design, Vasoconstrictor agents

## Abstract

**Background:**

In randomized clinical controlled trials, the choice of usual care as the comparator may be associated with better clinician uptake of the study protocol and lead to more generalizable results. However, if care processes evolve to resemble the intervention during the course of a trial, differences between the intervention group and usual care control group may narrow. We evaluated the effect on mean arterial pressure of an unblinded trial comparing a lower mean arterial pressure target to reduce vasopressor exposure, vs. a clinician-selected mean arterial pressure target, in critically ill patients at least 65 years old.

**Methods:**

For this multicenter observational study using data collected both prospectively and retrospectively, patients were recruited from five of the seven trial sites. We compared the mean arterial pressure of patients receiving vasopressors, who met or would have met trial eligibility criteria, from two periods: [1] at least 1 month before the trial started, and [2] during the trial period and randomized to usual care, or not enrolled in the trial.

**Results:**

We included 200 patients treated before and 229 after trial initiation. There were no differences in age (mean 74.5 vs. 75.2 years; *p* = 0.28), baseline Acute Physiology and Chronic Health Evaluation II score (median 26 vs. 26; *p* = 0.47) or history of chronic hypertension (*n* = 126 [63.0%] vs. *n* = 153 [66.8%]; *p* = 0.41). Mean of the mean arterial pressure was similar between the two periods (72.5 vs. 72.4 mmHg; *p* = 0.76).

**Conclusions:**

The initiation of a trial of a prescribed lower mean arterial pressure target, compared to a usual clinician-selected target, was not associated with a change in mean arterial pressure, reflecting stability in the net effect of usual clinician practices over time. Comparing prior and concurrent control groups may alleviate concerns regarding drift in usual practices over the course of a trial or permit quantification of any change.

**Supplementary Information:**

The online version contains supplementary material available at 10.1186/s12871-021-01529-w.

## Background

When an experimental intervention cannot be compared to placebo, researchers conducting randomized clinical trials have two options: protocolized or usual care control groups [1]. Usual care, also known as routine care, may be defined as the full spectrum of patient care processes and treatment decisions at the discretion of healthcare administrators or individual clinicians that may have an impact on patient outcomes [2]. Protocolized control groups reduce practice variability and may increase the signal-to-noise ratio, thus increasing the likelihood of observing a treatment effect [3]. However, this strategy poses a theoretical risk of comparing two interventions that may be inferior to usual care [4–6]. Alternatively, if practice changes over the course of the trial, the control group may become misaligned with usual care by the end of the trial [2, 3, 7]. Given these concerns, experts have questioned the conclusions drawn from randomized clinical trials that have changed practice in transfusion medicine and mechanical ventilation [5, 8, 9] and proposed usual care comparators as an alternative. This approach gives clinicians the freedom to provide what they consider to be optimal care in the control arm and eliminates the risk that an apparent benefit of an experimental intervention results from excess harm introduced in the control arm. However, usual care comparators allow more variability and, thus, may reduce the likelihood of observing a difference in clinical effect if one exists [3, 10].

Accordingly, when interpreting the result of a clinical trial with a usual care control arm, it is relevant to consider the extent to which the outcome of usual care in the trial corresponds to the outcome of usual care before the trial began. In 2018, we launched a clinical trial comparing a permissive hypotension strategy to reduce exposure to vasopressors vs. usual care in critically ill patients (NCT03431181) [11]. Vasopressors are medications given intravenously that increase blood pressure via vasoconstriction and that are commonly used liberally by clinicians despite their potential side effects [12–14]. The mean arterial pressure (MAP) in patients receiving vasopressors is most proximally vasopressor titration, although it may also be affected by other aspects of usual care including management of fluids, diuretics and ultrafiltration, and sedation. Concerned that the promotion of the trial and dissemination of background evidence at participating sites would raise awareness regarding the potential risks associated with usual care and modify standard practice during the trial, we compared mean arterial pressure (MAP) values in patients before vs. during the trial. A secondary objective was to compare MAP between three groups: patients treated with vasopressors before the initiation of the trial, patients in the trial’s control group, and patients who were potentially eligible for the trial while it was recruiting but who were not enrolled. Our hypothesis was that MAP would remain unchanged among these 3 groups of patients, reflecting stability in usual care practices.

## Methods

### Design

Multicenter observational study using data collected both prospectively and retrospectively to compare MAP of patients while receiving vasopressors before and during the Optimal VAsopressor TitratION in patients 65 years and older (OVATION-65) trial [11].

### Trial eligibility

**Fig. 1:**
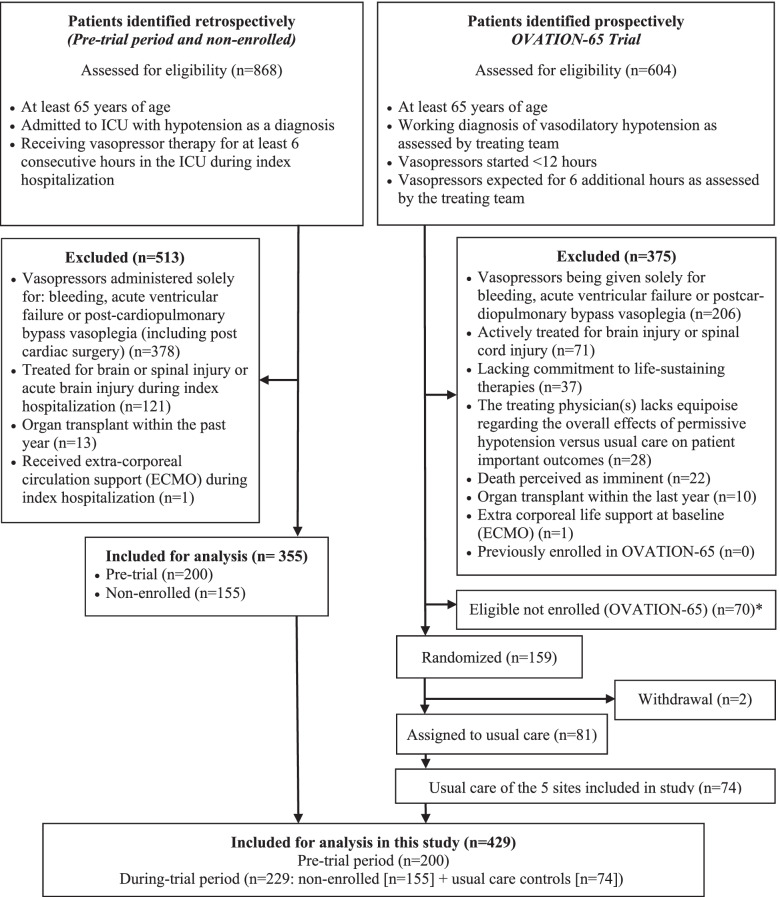
Patient’s flow chart – in the nested study. Abbreviations: ECMO, extracorporeal membrane oxygenation; ICU, intensive care unit; OVATION-65, Optimal VAsopressor TitratION in patients 65 years and older. * These patients were fully eligible but not enrolled in OVATION-65 and were not included in the analyses because identifying data, including medical record number, were not available

More information regarding the design of the OVATION-65 trial is available in the published protocol [11]. Briefly, the OVATION-65 trial enrolled 157 patients of 65 years of age or older who were receiving vasopressors for vasodilatory hypotension across seven Canadian sites (Fig. 1, Supplementary Table 1). Treating physicians had to confirm that patients were expected to receive vasopressors for at least six more hours. Other eligibility criteria appear in Table 1.

**Table 1:** Eligibility Criteria

PRE-TRIAL PERIOD	DURING-TRIAL PERIOD	
Pre-trial group	Non-enrolled group	Usual care control group
*Inclusion criteria*	*Inclusion criteria*	*Inclusion criteria*
•-At least 65 years of age •-Admitted to ICU with hypotension as a diagnosis •-Receiving vasopressor therapy for at least 6 consecutive hours in the ICU during index hospitalization	•-At least 65 years of age •-Admitted to ICU with hypotension as a diagnosis •-Receiving vasopressor therapy for at least 6 consecutive hours in the ICU during index hospitalization	•-At least 65 years of age •-Working diagnosis of vasodilatory hypotension as assessed by treating team •-Vasopressors started < 12 h before randomization •-Vasopressors expected for 6 additional hours as assessed by the treating team
*Exclusion criteria*	*Exclusion criteria*	*Exclusion criteria*
•-Treated for brain or spinal injury or acute brain injury during index hospitalization •-Vasopressors administered solely for: bleeding, acute ventricular failure or post-cardiopulmonary bypass vasoplegia •-Organ transplant within the past year •-Received ECMO during index hospitalization	•-Treated for brain or spinal injury or acute brain injury during index hospitalization •-Vasopressors administered solely for: bleeding, acute ventricular failure or post-cardiopulmonary bypass vasoplegia •-Organ transplant within the past year •-Received ECMO during index hospitalization	•-Actively treated for brain injury or spinal cord injury •-Vasopressors being given solely for bleeding, acute ventricular failure or post-cardiopulmonary bypass vasoplegia •-Lacking commitment to life-sustaining therapies •-Death perceived as imminent •-Previously enrolled in OVATION-65 •-Organ transplant within the last year •-ECMO at baseline •-The treating physician(s) lacks equipoise regarding the overall effects of permissive hypotension versus usual care on patient important outcomes

*Abbreviations*: *ECMO* extracorporeal membrane oxygenation, *OVATION-65* Optimal VAsopressor TitratION in patients 65 years and older, *ICU* intensive care unit

### Nested observational study eligibility

For this observational study, two investigators independently screened medical records to identify patients who would have met the OVATION-65 eligibility criteria 13 months to 1 month before the trial launched (‘pre-trial period’). They also identified patients who were potentially eligible for inclusion while the trial was ongoing but not enrolled (‘non-enrolled group’). The pre-trial and non-enrolled groups were both identified retrospectively from the list of all patients over 65 years old treated with vasopressors obtained from the medical records departments of participating sites. Of note, not all trial eligibility criteria could be applied retrospectively. For example, as a surrogate for the criterion of an anticipated duration of vasopressor therapy of at least six additional hours, we included patients who received vasopressors for 6 h or more (Table 1). The ‘during-trial period’ consisted of patients randomized in OVATION-65 to usual care (identified prospectively as the trial was ongoing) and the non-enrolled patients (identified retrospectively). We did not include patients identified prospectively as eligible for OVATION-65 but not enrolled (for example, because of lack of consent) because identifying details (including the medical record number) were not recorded for these patients.

### Measurements

For patients included in the trial, day 1 refers to the day of randomization whereas for patients in the other 2 groups, day 1 is defined as the day vasopressor therapy began. We recorded dose-rates for all vasopressor agents used (i.e. phenylephrine, epinephrine, vasopressin and dopamine) converting them to norepinephrine equivalents using a previously published formula [12, 15]. Severity of illness was assessed by the Acute Physiology and Chronic Health Evaluation II (APACHE II) score [16] in the 24 h following ICU admission. Patient’s MAP values while receiving vasopressors were collected hourly from day 1 until 24 h following discontinuation of vasopressors or day seven, whichever came first. Since blood pressure values are commonly measured multiple times per hour in ICUs, we used the values recorded closest to each hour mark, following the same instructions as in the OVATION-65 trial. We calculated the mean MAP while receiving vasopressors for each patient using all hourly recorded values while patients were receiving vasopressors (i.e. ignoring MAP values once vasopressors were discontinued).

In addition to actual MAP values, we collected from medical records hourly MAP targets - as prescribed by treating teams in the physician orders - as well as the following clinical outcomes: complications (supraventricular arrhythmia, ventricular arrhythmia, myocardial ischemia, clinically detected stroke, extremity ischemia, mesenteric ischemia and severe acute kidney injury, defined by stage 3 Kidney Disease Improving Global Outcomes criteria [17]), duration of ICU and hospital stay, ICU readmission(s), and ICU and hospital mortality. Patient’s characteristics at baseline (sex, age, comorbidities, admission type, reason of ICU admission, therapy at baseline, participating site and time from trial initiation to hospital admission of the patient) were also collected. We developed a case report form, created a detailed instructions manual, provided adequate training to the research personel involved in data collection and collected data in duplicate for 10% of the charts to ensure data accuracy and consistency. Clinicians involved in the management of vasopressor therapy were informed of the beginning of a trial less than 2 weeks before the site initiation visit and trained during the site initiation visit that corresponds to the start of the trial.

### Statistical analysis

#### Sample size

We included patients enrolled in the usual care control group of the OVATION-65 trial from five of the seven sites (2 sites lacked human resources to participate to this nested observational study; supplementary Table 1). In contrast, one site that was activated had still not enrolled a patient in the trial when enrollment was terminated but did collect retrospective data for this nested study. Data provided by this site were excluded of the adjusted sensitivity analysis. At the five sites that had enrolled a variable number of patients in the trial’s usual care control group, we collected data pertaining to a minimum of 30 patients [18] in each of the pre-trial and non-enrolled groups ensuring equal numbers were treated during the winter (November–April) and summer months (May–October) to account for potential seasonal variations in case-mix. The number of patients in the pre-trial and non-enrolled groups was increased at one site that enrolled more than 30 patients in the trial’s usual care arm. The statistical power of this sample size was over 95% and a difference of 2.5 mmHg in mean MAP could be detected.

#### Statistical analyses

Interrater agreement was evaluated for the selection of the patients identified retrospectively using a kappa statistic. Categorical data were reported as frequency (percent) and continuous data as mean (standard deviation [SD]) or median (interquartile range [IQR]) as appropriate. For comparisons of categorical variables between groups, Chi-square tests or Fisher’s exact test as dictated by the distribution of the data were used. For the primary analysis, we compared mean MAP of patients while receiving vasopressors in the pre-trial period and the during-trial period (including the non-enrolled and usual care control groups) using a Student T-Test. In a secondary analysis, using a multivariable linear regression model, the effect of trial initiation on mean MAP was measured and adjusted for the following prespecified independent variables chosen in function of their plausible impact on vasopressor management: age, chronic hypertension, APACHE II score, site, and time from trial initiation to hospital admission of the patient at each site. These variables were introduced simultaneously in the model. Sensitivity analyses, both adjusted and unadjusted, compared mean MAP values while receiving vasopressors across the three groups (i.e. pre-trial, non-enrolled, usual care controls).

Two sides *p* values less than 0.05 were considered statistically significant. We used SAS 9.4 (SAS Institute, Cary, NC, USA) for all analyses.

## Results

### Patients

Overall, 429 patients fulfilled the eligibility criteria and were included in this nested study: 74 from the trial’s usual care control group, 200 in the pre-trial group, and 155 in the non-enrolled group (Fig. 1, Supplementary Table 1). Agreement between reviewers for selection of patients in the pre-trial and non-enrolled groups was good (weighted kappa = 0.75, 95% confidence interval = 0.55–0.95) [19]. We initially identified a third party in case of disagreements but these were finally all resolved by consensus.

### Baseline characteristics and clinical outcomes

Table 2 presents patient characteristics by period and by group. No differences were noted for age (mean, pre-trial period: 74.5 [7.2] vs. during-trial period: 75.2 [6.9] years; *p* = 0.28) and APACHE II score (median, pre-trial period: 26 [20–31] vs during-trial period: 26 [21–31]; *p* = 0.47). During the trial period, men outnumbered women (during-trial period: *n* = 148 [64.6%] vs pre-trial period: *n* = 110 [55.0%]; *p* = 0.042), more patients received invasive ventilation (during-trial period: *n* = 144 [62.9%] vs pre-trial period: *n* = 102 [51%]; *p* = 0.013), and fewer received non-invasive ventilation (during-trial period: *n* = 50 [21.8%] vs pre-trial period: *n* = 75 [37.5%]; *p* = 0.0004). The mean MAP at the begining of data collection was similar between the two periods (mean, pre-trial period: 68.2 [13.7] vs during-trial period: 70 [13.1] mmHg; *p* = 0.16).

**Table 2:** Baseline Characteristics

Characteristic	*Usual care before* vs. *during the trial*		*Usual care pre-trial* vs. *non-enrolled* vs. *usual care controls*		
	Pre-trial period (***N*** = 200)	During-trial period (***N*** = 229)	Pre-trial group (***N*** = 200)	Non-enrolled group (***N*** = 155)	Usual care control group (***N*** = 74)
**Age,** years (mean [SD])	74.5 (7.2)	75.2 (6.9)	74.5 (7.2)	75.1 (6.8)	75.4 (7.2)
**Males** (n - %)	110 (55)	148 (64.6)	110 (55)	101 (65.2)	47 (63.5)
**APACHE II score** (median [IQR])	26 (20–31)	26 (21–31)	26 (20–31)	27 (22–31)	25.5 (20–29)
**Comorbidities** (n [%])					
*Cardiac*					
Supraventricular arrhythmia	48 (24)	66 (28.8)	48 (24)	47 (30.3)	19 (25.7)
Ventricular arrhythmia	0 (0)	3 (1.3)	0 (0)	3 (1.9)	0 (0)
Angina, MI, previous PCI, or CABG	51 (25.5)	63 (27.5)	51 (25.5)	55 (35.5)	8 (10.8)
CHF class I-III	24 (12.1)	42 (18.3)	24 (12.1)	32 (20.7)	10 (13.5)
CHF class IV	3 (1.5)	5 (2.2)	3 (1.5)	0 (0)	5 (6.8)
*Vascular*					
Chronic hypertension	126 (63)	153 (66.8)	126 (63)	102 (65.8)	51 (68.9)
Peripheral vascular disease or claudication	37 (18.5)	29 (12.7)	37 (18.5)	23 (14.8)	6 (8.1)
Cerebrovascular disease	19 (9.5)	40 (17.5)	19 (9.5)	22 (14.2)	18 (24.3)
*Endocrine*					
Diabetes	75 (37.5)	87 (38)	75 (37.5)	57 (36.8)	30 (40.5)
*Renal*					
Chronic dialysis	15 (7.5)	9 (3.9)	15 (7.5)	4 (2.6)	5 (6.8)
*Gastrointestinal*					
Moderate to severe liver disease	7 (3.5)	9 (3.9)	7 (3.5)	6 (3.9)	3 (4.1)
*Respiratory*					
Chronic lung disease	54 (27)	66 (28.8)	54 (27)	50 (32.3)	16 (21.6)
*Immunosuppression*					
Chemotherapy or chronic immunosuppressive	42 (21)	35 (15.3)	42 (21)	28 (18.1)	7 (9.5)
medications or transplantation					
*Neurologic*					
Cognitive impairment	16 (8)	16 (7)	16 (8.0)	14 (9)	2(2.7)
**Reason for ICU Admission** (n [%])					
*Medical*					
Cardiovascular/Vascular	3 (0.7)	10 (2.3)	3 (0.7)	9 (2.1)	1 (0.2)
Respiratory	38 (8.9)	52 (12.1)	38 (8.9)	40 (9.3)	12 (2.8)
Gastrointestinal	11 (2.6)	13 (3.0)	11 (2.6)	8 (1.9)	5 (1.2)
Sepsis	99 (23.1)	101 (23.5)	99 (23.1)	64 (14.9)	37 (8.6)
Other	8 (1.9)	8 (1.9)	8 (1.9)	3 (0.7)	5 (1.2)
*Surgical*					
Cardiovascular/vascular	4 (0.9)	3 (0.7)	4 (0.9)	3 (0.7)	0 (0)
Respiratory	14 (3.3)	6 (1.4)	14 (3.3)	6 (1.4)	0 (0)
Gastrointestinal	19 (4.4)	30 (7.0)	19 (4.4)	18 (4.2)	12 (2.8)
Other	4 (0.9)	6 (1.4)	4 (0.9)	4 (0.9)	2 (0.5)
**Life support at baseline** (n [%])					
Invasive ventilation	102 (51.0)	144 (62.9)	102 (51.0)	95 (61.3)	49 (66.2)
Non-invasive ventilation	75 (37.5)	50 (21.8)	75 (37.5)	33 (21.3)	17 (23)
Renal replacement therapy	17 (8.5)	10 (4.4)	17 (8.5)	6 (3.9)	4 (5.4)
**MAP at inclusion,** mmHg (mean [SD])	68.2 (13.7)	70 (13.1)	68.2 (13.7)	68.9 (14.4)	72.2 (9.7)
**Vasopressor dose-rate at inclusion,** ug/kg/min (mean [SD])	0.15 (0.23)	0.15 (0.18)	0.15 (0.23)	0.13 (0.17)	0.19 (0.19)

*Abbreviations*: *APACHE II* Acute Physiology and Chronic Health Evaluation II score, *CABG* coronary artery bypass grafting, *CHF* congestive heart failure, *ICU* intensive care unit, *IQR* interquartile range, *MAP* mean arterial pressure, *MI* myocardial infarction, *PCI* percutaneous coronary intervention, *SD* standard deviation

Clinical outcomes were similar across study periods and groups, including hospital mortality (pre-trial period: *n* = 75 [37.5%] vs during-trial: *n* = 78 [34.1%]; *p* = 0.46) and hospital length of stay (median, pre-trial period: 13.7 [7.3–25.4] vs during-trial period: 12.9 [7.0–25.1] days; *p* = 0.46) (Table 3).

**Table 3:** Mean arterial pressure, vasopressor use, and clinical outcomes

Variable	*Usual care before* vs. *during the trial*			*Usual care pre-trial* vs. *non-enrolled* vs. *usual care controls*			
	Pre-trial period (***N*** = 200)	During-trial period (***N*** = 229)	***p***-value	Pre-trial group (***N*** = 200)	Non-enrolled group (***N*** = 155)	Usual Care control group (***N*** = 74)	***p***-value
***Mean Arterial Pressure (MAP)***							
**MAP while receiving vasopressors** (mean [SD])	72.5 (5.1)	72.4 (5)	0.76	72.5 (5.1)	72.3 (5.1)	72.5 (4.9)	0.92
**MAP prescribed** (mean [SD])	64.7 (3.1)	65.3 (3.7)	0.08	64.7 (3.1)	65.1 (3.3)	65.5 (4.5)	0.15
***Vasopressor use***							
**Vasopressor duration,** hours (mean [SD])	48.5 (44.3)	43.6 (40.5)	0.24	48.5 (44.3)	45.3 (41.1)	40.3 (39.2)	0.36
**Vasopressor dose-rate,** μg/kg/min (mean [SD])	0.21 (0.3)	0.23 (0.37)	0.53	0.21 (0.3)	0.25 (0.41)	0.2 (0.27)	0.47
**Vasopressor total dose,** mg (mean [SD])	60.1 (122)	56.6 (104.9)	0.75	60.1 (121.9)	62.9 (118.6)	43.4 (66.5)	0.45
**Vasopressor(s) restarted,** n (%)	22 (11)	36 (15.7)	0.15	22 (11)	26 (16.8)	10 (13.5)	0.29
***Clinical Outcomes***							
**ICU Stay,** days (median [IQR])	4.0 (2.2–8.4)	5.0 (2.6–9.0)	0.43	4.0 (2.2–8.4)	5.1 (2.3–8.6)	5.3 (3.0–9.8)	0.24
**Hospital Stay,** days (median [IQR])	13.7 (7.3–25.4)	12.9 (7.0–25.1)	0.46	13.7 (7.3–25.4)	12.6 (6.6–24.1)	14.3 (8.5–28.1)	0.47
**ICU readmission(s),** n (%)	13 (6.5)	20 (8.7)	0.39	13 (6.5)	11 (7.1)	9 (12.2)	0.28
**Patients with ≥1 complication,** n (%)	88 (44.0)	98 (42.8)	0.80	88 (44.0)	73 (47.1)	25 (33.8)	0.16
**Mortality (ICU),** n (%)	52 (26.0)	66 (28.8)	0.51	52 (26.0)	46 (29.7)	20 (27.0)	0.74
**Mortality (Hospital)**, n (%)	75 (37.5)	78 (34.1)	0.46	75 (37.5)	54 (34.8)	24 (32.4)	0.71

*Abbreviations*: *MAP* mean arterial pressure, *ICU* intensive care unit, *IQR* interquartile range, *SD* standard deviation

### Mean MAP and vasopressor use among periods and groups

**Fig. 2:**
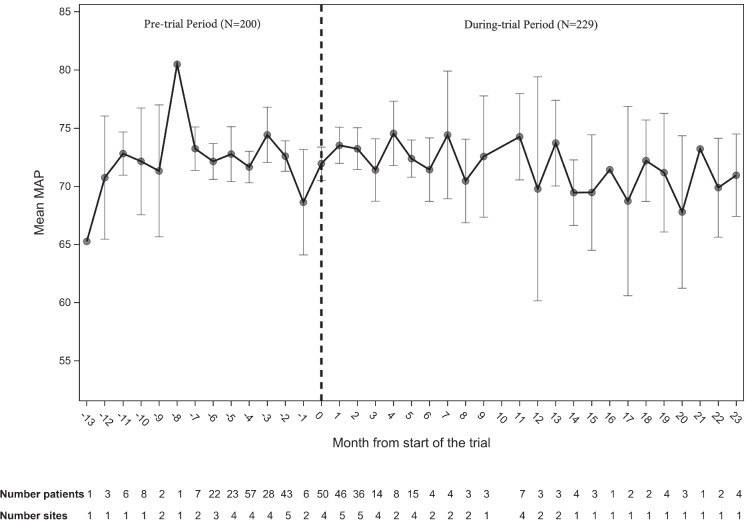
Mean arterial pressure over time. The *x* axis indicates the months from trial initiation. The *y* axis represents mean MAP. The dotted line indicates the trial initiation at each site, which was a different date at each site, and separates the pre-trial period, *N* = 200 (months − 13 to − 1) of the during-trial period, *N* = 229 (months 0–23). The number of patients included and the number of participating sites for each month are specified at the bottom of the figure

Mean MAP while receiving vasopressors was 72.5 (5.1) in the pre-trial period vs 72.4 (5.0) mmHg in the during-trial period (*p* = 0.76; Table 3). Figure 2 shows the overall mean MAP while receiving vasopressors by month relative to trial initiation across all sites. The mean prescribed target MAP (mean, pre-trial period: 64.7 [3.1] vs. during-trial period: 65.3 [3.7] mmHg; *p* = 0.08), duration of vasopressor therapy (mean, pre-trial period: 48.5 [44.3] vs. during-trial period: 43.6 [40.5] hours; *p* = 0.24), and total vasopressor dose (mean, pre-trial period: 60.1 [122] vs. during-trial period: 56.6 [104.9] mg norepinephrine equivalents; *p* = 0.75) were similar between periods. Comparing between 3 groups, we found no difference in mean MAP while receiving vasopressors (mean, pre-trial group: 72.5 [5.1] vs non-enrolled group: 72.3 [5.1], vs. usual care control group: 72.5 [4.9] mmHg; *p* = 0.92).

Adjusted analyses did not reveal an association between trial initiation and mean MAP while receiving vasopressors (Table 4) but suggested that higher MAP values were achieved in one of the 5 participating sites.

**Table 4:** Multivariable linear regression evaluating association between MAP values while receiving vasopressors and trial initiation

*Trial initiation defined by a dichotomous variable (pre-trial* vs. *during-trial periods)*				*Trial initiation defined by a three-category variable (pre-trial period, non-enrolled group, usual care control group)*			
Variable	β coefficient	95% Confidence interval	*p*-value	Variable	β coefficient	95% Confidence interval	*p*-value
**During-trial period** **(Non-enrolled + Usual care control groups)**	**0.47**	**(− 0.85, 1.80)**	**0.48**	**Non-enrolled group**	**0.66**	**(− 0.68, 2.00)**	**0.33**
				**Usual care control group**	**2.35**	**(−0.11, 4.82)**	**0.06**
Pre-trial period	Reference			Pre-trial period	Reference		
APACHE II, per 10-point increase	−0.07	(−0.13, − 0.01)	0.03	APACHE II, per 10-point increase	− 0.06	(− 0.12, − 0.001)	0.04
Chronic hypertension (yes)	−0.71	(−1.72, 0.31)	0.17	Chronic hypertension (yes)	−0.71	(−1.72, 0.31)	0.17
Age, per 5-year increase	−0.03	(−0.1, 0.03)	0.32	Age, per 5-year increase	−0.05	(−0.12, 0.02)	0.18
Site 1	−1.22	(−2.61, 0.16)	0.08	Site 1	−1.11	(−2.49, 0.27)	0.11
Site 2	−1.32	(−2.96, 0.31)	0.11	Site 2	−1.32	(−2.94, 0.30)	0.11
Site 3	1.01	(−0.66, 2.68)	0.24	Site 3	1.17	(−0.49, 2.83)	0.17
Site 5	5.63	(2.05, 9.22)	0.002	Site 4	Reference		
Site 4	Reference						
Time from trial initiation to hospital admission	−0.001	(−0.005, 0.002)	0.39	Time from trial initiation to hospital admission	−0.004	(−0.009, 0.0004)	0.07

The 2-group analysis includes 429 patients from five sites. The three-group analysis include 419 patients from four sites, because the fifth site did not enroll any patients in the trial. Abbreviations: APACHE II, Acute Physiology and Chronic Health Evaluation II score

## Discussion

In this nested observational study, the initiation of a trial of permissive hypotension to reduce vasopressor exposure did not impact the MAP achieved at the participating sites, reflecting stability of the net effect of usual care processes before and during the trial. Reasurance that usual care received by patients in the control arm of the trial was similar to usual care before trial initiation and also to usual care as delivered to patients who were not enrolled but treated during the trial will enrich the interpretation of the trial results. The approach consisting of ascertaining potential fluxes in usual care over time and across concurrent control groups would be applicable to other research settings and may help readers of clinical trial reports evaluate the applicability of future trial results.Despite methodologic commentary on the importance of characterizing usual care when compared to an experimental intervention, we were unable to find any study measuring the impact of the initiation of a randomized controlled trial on usual care [1–4, 7].

Multivariable analysis showed that trial initiation was not associated with change in achieved MAP, but when the post-trial period was divided into patients randomized to usual care in OVATION-65 and non-enrolled patients, the association between usual care control group in OVATION-65 (vs. pre-trial period) and higher achieved MAP was almost statistically significant. This finding may be related to chance, due to a smaller sample size in the usual care control group and greater potential for influential outliers. Alternatively, the finding may reflect residual confounding from differences in severity of illness, or potentially an influence of trial initiation on care practices. However, the direction of effect, if related to the initation of OVATION-65, is opposite to our concern that the trial would have led to lower achieved MAP. Therefore, spurious finding or residual confounding are more likely explanations.

The study exhibits the following strengths. The sample size was sufficiently large to discern differences in the continuous outcome of MAP. We planned a one-month washout period separating the pre-trial and during-trial periods to minimize contamination between these two periods. Data collection that purposefully spanned winter and summer months, to mitigate the potential effects of seasonal case mix variations is also an important strength. In addition, the fact that mean MAP values were consistent with the results of a previously reported observational study published in 2017 reinforces the plausibility of these contemporaneous observations [13].

This study also has limitations. Although patients across study periods presented similar characteristics, small differences in the distribution of certain comorbidities, such as coronary disease, heart failure, and stroke, were observed and may be owed to the relatively small sample size. Mean MAP was numerically lower in the pre-trial and non-enrolled groups, likely because data collection for those patients started from vasopressor initiation. In contrast, for patients in the trial’s usual care control group, data collection started after randomization, at which time patients had already been partially stabilized. In the multivariable linear regression analyses, a higher severity of illness was associated with a lower mean MAP while receiving vasopressors, which may reflect a more severe cardiovascular compromise. The fact that MAP values varied by site reflects fluctuations in local cultures that have been previously described and is common when the certainty of evidence guiding clinical care is low and subject to interpretation [20]. One site contributed 46% of the data for this analysis, which may have masked the impact of trial initiation at other sites. Moreover, two of seven trial sites could not participate to this nested study, and of the five participating sites, one did not enroll patients in the trial. Although this limitation justifies some caution before concluding that the results apply equally across sites, the amount of data collected at each site appropriately reflects the number of patients enrolled from each site in the original trial. Patients in the trial were similar, but not identical, to those identified retrospectively, given the impossibility of retrospectively operationalizing the trial critierion of expected future duration of vasopressor therapy of at least 6 h. Finally, the mean arterial pressure values recorded in this study were similar to those measured in previous observational studies [13, 20], suggesting that foreknowledge of the launch of OVATION-65 was unlikely to have influenced practice in these centres.

## Conclusion

The initiation of an unblinded trial comparing a permissive hypotension strategy to usual care in critically ill patients did not impact usual care in the centres where the trial was conducted. The approach consisting of ascertaining potential fluxes in usual care over time and across concurrent control groups would be applicable to other research settings and may alleviate concerns regarding potential biases or permit quantification of said biases.

## Supplementary Information

**Additional file 1: Supplement Table 1.** Number of Patients included per site.

## Data Availability

The datasets used and/or analysed during the current study are available from the corresponding author on reasonable request.

## Abbreviations

APACHE II: Acute Physiology and Chronic Health Evaluation II
CABG: Coronary artery bypass grafting
CHF: Congestive heart failure
ECMO: Extracorporeal membrane oxygenation
ICU: Intensive Care Unit
IQR: Interquartile range
MAP: Mean arterial pressure
MI: Myocardial infarction
NA: Not applicable
OVATION-65: Optimal VAsopressor TitratION in patients 65 years and older
PCI: Percutaneous coronary intervention
SD: Standard deviation

## References

[1] Silverman HJ, Miller FG (2004). Control group selection in critical care randomized controlled trials evaluating interventional strategies: an ethical assessment. Crit Care Med.

[2] Thompson BT, Schoenfeld D (2007). Usual care as the control group in clinical trials of nonpharmacologic interventions. Proc Am Thorac Soc.

[3] Angriman F, Masse MH, Adhikari NKJ (2019). Defining standard of practice: pros and cons of the usual care arm. Curr Opin Crit Care.

[4] Deans KJ, Minneci PC, Danner RL, Eichacker PQ, Natanson C (2010). Practice misalignments in randomized controlled trials: identification, impact, and potential solutions. Anesth Analg.

[5] Times N (2018). Trial by Fire: Critics Demand That Huge Sepsis Study Be Stopped.

[6] Takala J (2009). Better conduct of clinical trials: the control group in critical care trials. Crit Care Med.

[7] Lantos JD, Feudtner C (2015). SUPPORT and the ethics of study implementation: lessons for comparative effectiveness research from the trial of oxygen therapy for premature babies. Hast Cent Rep.

[8] Hebert PC, Wells G, Blajchman MA, Marshall J, Martin C, Pagliarello G (1999). A multicenter, randomized, controlled clinical trial of transfusion requirements in critical care. Transfusion requirements in critical care investigators, Canadian critical care trials group. N Engl J Med.

[9] Brower RG, Matthay MA, Morris A, Schoenfeld D, Thompson BT, Wheeler A (2000). Ventilation with lower tidal volumes as compared with traditional tidal volumes for acute lung injury and the acute respiratory distress syndrome. N Engl J Med.

[10] Arch JJ, Stanton AL (2019). Examining the “usual” in usual care: a critical review and recommendations for usual care conditions in psycho-oncology. Support Care Cancer.

[11] Masse MH, Battista MC, Wilcox ME, Pinto R, Marinoff N, D'Aragon F (2020). Optimal VAsopressor TitraTION in patients 65 years and older (OVATION-65): protocol and statistical analysis plan for a randomised clinical trial. BMJ Open.

[12] Lamontagne F, Richards-Belle A, Thomas K, Harrison DA, Sadique MZ, Grieve RD (2020). Effect of reduced exposure to vasopressors on 90-Day mortality in older critically ill patients with vasodilatory hypotension: a randomized clinical trial. JAMA.

[13] Lamontagne F, Cook DJ, Meade MO, Seely A, Day AG, Charbonney E (2017). Vasopressor use for severe hypotension-a multicentre prospective observational study. PLoS One.

[14] Andreis DT, Singer M (2016). Catecholamines for inflammatory shock: a Jekyll-and-Hyde conundrum. Intensive Care Med.

[15] Khanna A, Ostermann M, Bellomo R (2017). Angiotensin II for the treatment of vasodilatory shock. N Engl J Med.

[16] Knaus WA, Draper EA, Wagner DP, Zimmerman JE (1985). APACHE II: a severity of disease classification system. Crit Care Med.

[17] Disease K (2012). Improving Global Outcomes (KDIGO) Acute Kidney Injury Work Group. KDIGO Clinical Practice Guideline for Acute Kidney Injury. Kidney Int Suppl.

[18] Kwak SG, Kim JH (2017). Central limit theorem: the cornerstone of modern statistics. Korean J Anesthesiol.

[19] Byrt T (1996). How good is that agreement?. Epidemiology.

[20] St-Arnaud C, Ethier JF, Hamielec C, Bersten A, Guyatt G, Meade M (2013). Prescribed targets for titration of vasopressors in septic shock: a retrospective cohort study. CMAJ Open.

