# Supplementary material for: The evolution of mean arterial pressure in critically ill patients on vasopressors before and during a trial comparing a specific mean arterial pressure target to usual care
Source: BMC Anesthesiol. 2022 Jan 3;22:6. doi: 10.1186/s12871-021-01529-w (PMC8722048; doi:10.1186/s12871-021-01529-w)
Supplement: Supplementary file 1 — Additional file 1: Supplement Table 1. Number of Patients included per site. [file 12871_2021_1529_MOESM1_ESM.docx]

#### Supplement Table 1 Number of Patients included per site

| **OVATION-65 Participating Sites** | **Pre-trial period** | **During-trial period** | | **Total** |
| --- | --- | --- | --- | --- |
|  | **Pre-trial group** | **Eligible not enrolled group** | **Usual Care controls group** |  |
| Site 1 | 90 | 60 | 50 | 200 |
| Site 2 | 35 | 30 | 7 | 72 |
| Site 3 | 35 | 30 | 6 | 71 |
| Site 4 | 35 | 30 | 11 | 76 |
| Site 5 | 5 | 5 | 0 | 10 |
| Site 6 | Could not participate to nested  observational study | | 4* | NA |
| Site 7 |  |  | 3* | NA |
| TOTAL | 200 | 155 | 74 | 429 |

NA: not applicable

*Patients not included in this nested observational study
